# The mechanism of action of clozapine

**DOI:** 10.1177/02698811251319458

**Published:** 2025-02-13

**Authors:** Paul D Morrison, Sameer Jauhar, Allan H Young

**Affiliations:** 1 NHS Highland Health and Social Care Partnership, Lochgilphead, Scotland; 2 Institute of Psychiatry, Psychology, and Neuroscience, King’s College London, London, UK; 3 South London and Maudsley NHS Foundation Trust, Beckenham, Kent

**Keywords:** Schizophrenia, psychosis, clozapine, mechanism, acetylcholine, cholinergic, muscarinic, agonist, M_4_, M_1_, receptor, striatum, xanomeline, KarXT, Cobenfy, emraclidine

## Abstract

Previous hypotheses for the superiority of clozapine over other antipsychotics have failed to stand the test of time. Here we describe how the unique pharmacology of clozapine in the peripheral nervous system held clues for solving the puzzle of clozapine in the central nervous system. Clozapine appears to have been the prototype for a new class of antipsychotics, now entering clinical psychiatry, which activates muscarinic acetylcholine receptors.

## Introduction: The unique clinical pharmacology of clozapine

In comparison to the other antipsychotics, clozapine has a unique clinical pharmacology. Beyond doubt, clozapine has efficacy in treatment-resistant schizophrenia (Wagner et al., 2021). In contrast with many other antipsychotics, clozapine is free from motor side effects. Finally, the benefits of clozapine for schizophrenia extend to a lower suicide rate and a lower mortality rate compared to other antipsychotic molecules, or no treatment (Forte et al., 2021; Wagner et al., 2021).

A longstanding question for the field has been as follows: what is the nature of the underlying receptor pharmacology that differentiates clozapine from the other antipsychotics? Two influential hypotheses have been proposed to account for the distinct efficacy of clozapine, specifically that clozapine has a unique binding pattern at central dopamine receptors or that it binds to serotonin 5HT_2A_ receptors, but both proposals have their shortcomings (Nucifora et al., 2017).

One clue was perhaps missed all along. Clozapine has a unique clinical pharmacology in the autonomic nervous system. Whereas numerous antipsychotics, particularly phenothiazines such as chlorpromazine inhibit autonomic nervous system responses, clozapine, in stark contrast, can stimulate autonomic responses. For example, in salivary gland tissue, clozapine produces excessive salivation, whereas many other antipsychotics elicit the exact opposite effect, a dry mouth, or have no discernible effect.

Hypersalivation is the most common side effect of clozapine, occurring in over 90% of patients (Maher et al., 2016; Sanagustin et al., 2023). For decades, it has been known that salivation is evoked by the neurotransmitter acetylcholine acting at muscarinic receptors (Brown, 2006). Clozapine is distinct among antipsychotic drugs in being able to stimulate muscarinic receptors in the salivary gland (Ishikawa et al., 2020).

Stimulation of muscarinic receptors in the brain was proposed as the mechanism accounting for the unique antipsychotic effects of clozapine (Morrison et al., 2020), based in part, on the observation that a muscarinic agonist had tested positive in an assay which, since the time of chlorpromazine, has been used to discover antipsychotic drugs, the conditioned avoidance response (Watt et al., 2013). One subtype of muscarinic receptor stood out, the M_4_ receptor, a theme to which we will return.

## The unique receptor pharmacology of clozapine: Not dopamine

Since the 1970s, the antagonism of dopamine D_2_ receptors has been central to the antipsychotic story. The site of action is believed to be within the microcircuitry of the striatum (Morrison and Murray, 2018). More recently, it has been proposed that, in schizophrenia, there is an excess of dopamine within the nerve fibres projecting into the striatum (Howes and Murray, 2014; Jauhar et al., 2022).

At significant odds with the dopamine story, however, clozapine has a low affinity for the dopamine D_2_ receptor. This has been found in vitro using cloned human D_2_ receptors, and in patients with schizophrenia using neuroreceptor imaging techniques (Pilowsky et al., 1992). Clozapine is similar to quetiapine, in terms of having low affinity for the D_2_ receptor, whereas the other commonly used antipsychotics have an affinity for D_2_ which is often several orders of magnitude higher (Table 1). While low affinity for the dopamine D_2_ receptor can explain why clozapine and quetiapine are free of motor side effects (Kapur and Seeman, 2001), it cannot account for superior efficacy, as clozapine outperforms quetiapine as an antipsychotic (Nucifora et al., 2017).

**Table 1.** The affinity of commonly prescribed and novel drugs for receptors associated with antipsychotic activity. Binding to dopamine D_2_ and serotonin 5HT_2A_ does not differentiate clozapine from other the other antipsychotic drugs, and therefore cannot be the basis of the distinct efficacy of clozapine. Clozapine differs from the other antipsychotic drugs at muscarinic receptors, in that it functions as a partial agonist. The M_4_ receptor has attracted particular interest, in light of preclinical work showing that M_4_ agonism carries an antipsychotic signature. Xanomeline and emraclidine also share clozapine’s ability to stimulate muscarinic M_4_ receptors. Clozapine is thus the prototype of a new class of antipsychotic molecules, whose mechanism of action is agonism at cholinergic muscarinic receptors.

Antipsychotic drug	Dopamine D_2_	Serotonin 5HT_2A_	Cholinergic M_4_
Receptor affinity			
Aripiprazole	++++Partial agonist	++	0
Haloperidol	+++	++	0
Risperidone	+++	++++	0
Chlorpromazine	+++	+++	++
Olanzapine	++	+++	+
Quetiapine	+	++	+
Clozapine	+	+++	++Partial agonist (M_4_)
Xanomeline and trospium (KarXT)	0	+/0	++Partial agonist (M_1_ and M_4_)
Emraclidine	No data	No data	+++Partial allosteric modulator (M_4_)

Source: Data from Besnard et al. (2012), Krystal et al. (2022).

Affinity: ++++ very high (<1 nm), +++ high (1–10 nM), ++ moderate (10–100 nM), + low (100–1000 nM), 0 non-significant (>1000 nM).

Clozapine has a higher affinity for D_4_ compared to D_2_ dopamine receptors, which led to the proposal that D_4_ binding accounted for clozapine’s distinct efficacy. The idea was short-lived, however, with the failure of selective D_4_ antagonists in clinical trials, and it was already apparent that many other antipsychotics also block D_4_ receptors (Nucifora et al., 2017). A similar story played out at the dopamine D_1_ receptor with the failure of a selective D_1_ antagonist in patients with schizophrenia (Karlsson et al., 1995).

## The unique receptor pharmacology of clozapine: Not serotonin

The serotonin story in schizophrenia begins with the ability of lysergic acid diethylamide (LSD), psilocybin and other psychedelic molecules mimic the experience of psychosis in healthy brains (Colpaert, 2003). The psychedelics produce their effects by stimulation of central 5HT_2A_ receptors (López-Giménez and González-Maeso, 2018).

There was an effort to develop LSD antagonists as possible antipsychotics (Colpaert, 2003). Risperidone, the first to the clinic (Colpaert, 2003), has a very high affinity for the 5HT_2A_ receptor but fails to match clozapine in treatment-resistant schizophrenia. The same is true for other antipsychotics of the so-called dual D_2_/5HT_2A_ class, of which risperidone was the prototype molecule (Nucifora et al., 2017). Certainly, clozapine has a high affinity for the 5HT_2A_ receptor, but this feature is not unique within either the first- or second-generation class of antipsychotics. As such, binding to 5HT_2A_ receptors is unlikely to account for the distinct efficacy of clozapine (Nucifora et al., 2017).

A footnote to the serotonin story had, for a period, reinvigorated enthusiasm for 5HT_2A_ antagonists (and inverse agonists) in psychosis. In 2016, pimavanserin, which binds to 5HT_2A_ but not dopamine D_2_ receptors (‘*a pure*’ 5HT_2A_ drug), gained FDA approval for the treatment of psychotic symptoms in Parkinson’s disease. Unfortunately, trials of pimavanserin in schizophrenia have been disappointing. Early hopes of efficacy against negative symptoms failed to materialise in a definitive, phase III trial (ADVANCE-2), and the manufacturer has withdrawn pimavanserin from further development in schizophrenia. To date, a similarly disappointing scenario has occurred with another selective 5HT_2A_ drug, roluperidone.

## Acetylcholine: A primer

Acetylcholine is the prototypical neurotransmitter, characterised in the early 20th century as the chemical intermediate between the vagus nerve and the heart, and the transmitter at the neuromuscular junction (Brown, 2006). Acetylcholine mediates fast transmission via nicotinic receptor channels, and slow transmission via G-protein-coupled receptors of the muscarinic class, isolated, cloned and sequenced as M_1_–M_5_ subtypes in the 1980s (Brown, 2019), and structurally resolved in the last 12 years (Tobin, 2024).

Muscarinic M_1_–M_5_ receptors are found in the brain as well as the periphery. The main subtypes expressed in the brain are M_1_, enriched in the cortex, striatum and hippocampus and M_4_, enriched in the striatum. In basic terms, M_1_ receptors are post-synaptic and excitatory, whereas M_4_ receptors are localised to nerve terminals, and inhibit neurotransmitter release (Brown, 2019).

Cholinergic pathways in the CNS are well-demarcated. They can be grouped into three categories. Firstly, cholinergic neurons are involved in new learning and memory project from basal forebrain centres to the cortex and hippocampus. Secondly, cholinergic fibres originating from the pedunculopontine and laterodorsal tegmental nuclei of the brainstem join the ascending arousal system. Notably, these cholinergic fibres also ascend to dopamine centres in the midbrain. Finally, there is a population of cholinergic interneurons within the striatum, which synapse on medium spiny neurons, the output neuron of the striatum (Nieuwenhuys et al., 2008).

## The unique receptor pharmacology of clozapine: Acetylcholine

Numerous first- and second-generation antipsychotics, clozapine included, bind to muscarinic receptors within the nanomolar range (Besnard et al., 2012). But the key difference is that, whereas many other antipsychotics are potent antagonists at muscarinic receptors, clozapine functions as an agonist, as observed in salivary gland tissue, evoking a dry mouth, or hypersalivation, respectively. Norclozapine, the major metabolite of clozapine, is a potent agonist at M_1_ receptors and mediates hypersalivation (Ishikawa et al., 2020), but it lacks antipsychotic activity of its own. Clozapine is a partial agonist at M_4_ receptors (Olianas et al., 1997; Zorn et al., 1994).

A decisive test emerges, which is whether an agonist at muscarinic M_4_ receptors, but with little affinity for dopamine D_2_ receptors, exhibits antipsychotic properties, not just in preclinical assays as described above (Watt et al., 2013), but in people with schizophrenia.

This has now been clarified. Phase III clinical trials of the pharmaceutical KarXT in schizophrenia have been positive (Kaul et al., 2024a, 2024b). KarXT has since won regulatory approval for the treatment of schizophrenia in the USA and will be marketed as Cobenfy. KarXT is composed of an agonist with some selectivity for M_1_ and M_4_ within the overall muscarinic class (xanomeline) but which still requires, for tolerability, the inclusion of a broad spectrum, non-brain-penetrant muscarinic antagonist (trospium) to offset peripheral autonomic side effects (Paul et al., 2022).

Emraclidine, a selective positive allosteric modulator at M_4_ receptors, has now progressed to late-stage clinical trials in schizophrenia (Krystal et al., 2022). Emraclidine is one of eight cholinergic candidates in development for cognition (M_1_) or psychosis (M_4_) (Tobin, 2024), early refinements to what already looks like a new era in psychopharmacology with the success of KarXT.

Some key clinical questions may be answered in the next few years. Firstly, are the new cholinergic drugs effective in treatment-resistant psychosis? Secondly, do they have convincing efficacy against the primary negative and cognitive symptoms of schizophrenia? Thirdly, do they, like clozapine (Mentzel et al., 2018), improve tardive dyskinesia? Fourthly, do they reduce the rates of suicide and mortality to the same extent as clozapine? Finally, are the new cholinergic drugs free of the severe adverse drug reactions associated with clozapine, such as pneumonia, agranulocytosis, myocarditis, weight gain and ileus? The findings from the recent phase III trials of KarXT have been encouraging with favourable tolerability, an absence of weight gain and relatively mild gastrointestinal side effects (Kaul et al., 2024a, 2024b).

It will also be interesting to observe new insights into dopamine/acetylcholine crosstalk in the basal ganglia and if, for example, an omnibus effect at D_2_, M_4_ and M_1_ emerges as an important factor.

## Conclusion

Clozapine has a unique clinical pharmacology, compared to the other antipsychotics. The long-sought mechanism of action for clozapine’s distinct antipsychotic properties appears to be partial agonism at muscarinic acetylcholine receptors (M_4_ and possibly M_1_).

There had been clues from peripheral autonomic pharmacology, where clozapine and its metabolite norclozapine, in stark contrast to the other antipsychotics, can stimulate tissue responses mediated via muscarinic receptors.

Agonists at muscarinic receptors have efficacy in schizophrenia, having previously shown promise in the conditioned avoidance response, an important marker of antipsychotic activity. To date, one cholinergic-based pharmaceutical, other than clozapine, KarXT, has translated to clinical practice. Other cholinergic molecules may follow in the near future.

With the emergence of cholinergic-based antipsychotics, some have observed that we are at a major milestone in psychiatry, similar to Delay and Deniker’s demonstration of the antipsychotic effects of chlorpromazine in the 1950s, or indeed, the introduction of clozapine itself in the latter decades of the 20th century. It would be an irony if the clarification of the mechanism of action of clozapine occurred alongside the emergence of drugs which could potentially supersede it in clinical practice.
